# Effect of Sulfites on Antioxidant Activity, Total Polyphenols, and Flavonoid Measurements in White Wine

**DOI:** 10.3390/foods7030035

**Published:** 2018-03-09

**Authors:** Mirella Nardini, Ivana Garaguso

**Affiliations:** 1 CREA Research Centre for Food and Nutrition, via Ardeatina 546, 00178 Rome, Italy; 2 CREA Research Centre for Plant Protection and Certification, via C.G. Bertero 22, 00156 Rome, Italy; ivana.garaguso@crea.gov.it

**Keywords:** wine, polyphenols, flavonoids, antioxidant activity, sulfites, polyvinylpyrrolidone

## Abstract

Polyphenols content and antioxidant activity are directly related to the quality of wine. Wine also contains sulfites, which are added during the winemaking process. The present study aimed to evaluate the effects of sulfites on the assays commonly used to measure the antioxidant activity and polyphenols and flavonoids content of white wines. The effects of sulfites were explored both in the standard assays and in white wine. The addition of sulfites (at 1–10 μg) in the standard assays resulted in a significant, positive interference in the Folin–Ciocalteu’s assay used for polyphenols measurements and in both the Ferric Reducing Antioxidant Power and 2,2′-azino-bis (3-ethylbenzothiazoline-6-sulfonic acid) diammonium salt radical cation decolorization assays, which were used for antioxidant activity evaluation. A negative interference of sulfites (at 1–20 μg) was observed for the colorimetric aluminium-chloride flavonoids assay. The addition of sulfites to organic white wines (at 25–200 mg/L wine) clearly resulted in a significant overestimation of antioxidant activity and polyphenols content, and in an underestimation of flavonoids concentration. To overcome sulfite interferences, white wines were treated with cross-linked polyvinylpyrrolidone. The total polyphenols content and antioxidant activity measurements obtained after polyvinylpyrrolidone treatment were significantly lower than those obtained in the untreated wines. Flavonoids were expected to be higher after polyvinylpyrrolidone treatment, but were instead found to be lower than for untreated wines, suggesting that in addition to sulfites, other non-phenolic reducing compounds were present in white wine and interfered with the flavonoid assay. In view of our results, we advise that a purification procedure should be applied in order to evaluate the quality of white wine.

## 1. Introduction

Dietary antioxidants may offer protection against oxidative stress-related diseases, such as atherosclerosis, diabetes, neurodegenerative diseases, aging, and cancer [1]. Polyphenols are by far the most abundant antioxidants in most diets [1,2]. Epidemiological evidence indicates that long-term consumption of polyphenol-rich foods and beverages is inversely associated with the risk of developing oxidative stress-related diseases [1,3].

For individuals regularly consuming wine, coffee, beer, fruit juice, and tea, these beverages will be the major sources of phenolics antioxidants [4]. Among beverages, wine has been recognized to have beneficial effects on human health when drunk in moderation. Hypolipidaemic, hypotensive, and anti-atherosclerotic effects, antioxidant status improvement, and reductions in oxidation biomarkers have been described in human intervention studies [5,6,7]. Several epidemiological studies reported significant reductions in all-cause and particularly cardiovascular mortality in moderate wine drinkers when compared to abstainers or to individuals drinking excess alcohol [8,9,10].

The total amount of wine polyphenols has been estimated in the range of 800–6000 mg/L for red wines and 50–350 mg/L for white wines [11,12]. These compounds are directly related to the quality of wines and are responsible for most of the antioxidant activity of wines. Therefore, accurate total polyphenols content and antioxidant activity measurements are essential to evaluate wine quality and to characterize and compare different wines. The well-known Folin–Ciocalteu (FC) assay has been adopted by the European Commission for analysis of total polyphenols in wine [13,14,15,16]. The colorimetric aluminium-chloride assay is widely used to measure total flavonoids in wine [17,18]. The antioxidant activity of wine is usually measured by several methods: 2,2′-azino-bis (3-ethylbenzothiazoline-6-sulphonic acid (ABTS) radical cation assay; ferric reducing antioxidant power (FRAP) assay; 2,2-diphenyl-1-picrylhydrazyl (DPPH) radical scavenging assay; and oxygen radical absorbing capacity (ORAC) assay [4,19].

In addition to polyphenols, wine may also contain pesticides, preservatives (sulfur dioxide, sulfites), trace metals, neurotoxins, potential carcinogens, and allergens [20]. Among these, sulfur dioxide, sulfite, and metabisulfite (hereafter referred to as sulfites) are widely used at different stages of winemaking and storage for their sterilizing and antibacterial properties and to prevent phenolics oxidation, which adversely affects the sensory properties and nutritional value of wine [21]. Due to the risk to human health, the total sulfites content, expressed as sulfur dioxide, cannot exceed 150 and 200 mg/L for conventional red and white wines, respectively, and 100 and 150 mg/L in organic red and white wines, respectively [21].

Interference of sulfites and other non-phenolic reducing compounds in the FC assay, used for polyphenols determination in foods and beverages, particularly white wine, has occasionally been reported [15,22,23,24]. Recently, an analysis of white wine with the DPPH and FC assays has been found to overestimate the contribution of phenolics to the antioxidant activity [25]. Sulfite alone has been reported to react weakly in the ABTS assay [26]; however, the effect of sulfite on the reaction of polyphenols with the ABTS reagent has not been examined.

From data in the literature, it arises that a systematic study on the effects of sulfites on the analytical methods used to characterize the polyphenols content and antioxidant activity of wine has never been accomplished. This study aims to investigate the effects of sulfites on several methods commonly used for total polyphenols (FC assay), flavonoids (aluminium-chloride assay), and antioxidant activity (FRAP and ABTS assays), either in standard assays or in white wine. To overcome sulfite interference, the efficacy of cross-linked polyvinylpyrrolidone (PVPP) treatment of white wine was studied.

## 2. Materials and Methods

### 2.1. Materials

Gallic acid, (+) catechin hydrate, (±) 6-hydroxy-2,5,7,8-tetramethyl-chroman-2-carboxylic acid (Trolox), potassium peroxodisulfate, 2,4,6-tris(2-pyridyl)-S-triazine (TPTZ), Iron (III) chloride hexahydrate, 2,2′-azino-bis(3-ethylbenzothiazoline-6-sulfonic acid) diammonium salt (ABTS), ferrous sulfate, aluminium chloride hexahydrate, sodium sulfite, sodium metabisulfite, and Folin–Ciocalteu’s phenol reagent were from Sigma (St. Louis, MO, USA). Cross-linked polyvinylpyrrolidone (PVPP) was from Acros Organics (Morris Plains, NJ, USA). All other reagents were of the best grade available.

### 2.2. Wines

Both organic and conventional white wines were produced in Italy and purchased at local markets and wine shops. The characteristics of the wines are given in Table 1. All organic white wines were produced according to the official organic farming practices (Italian Association for Organic Farming, AIAB, Italy), which typically excludes the use of artificial chemical fertilizers, pesticides, fungicides, and herbicides, and these wines had certificates of organic production. The winemaking protocol used suited the requisites of the European Union (EU) regulation for organic wine production [27]. All of the organic white wines were obtained without sulfur dioxide/sulfites addition during winemaking processes. In a separate set of experiments, 10 mL aliquots of organic white wines produced without sulfites addition during winemaking were spiked with increasing amounts of sodium sulfite or sodium metabisulfite to give final concentrations in the range of 25–200 mg/L wine, and analyzed 15 min after addition. The addition rate used, i.e., 25–200 mg/L, was chosen to reproduce the sulfites concentrations present in conventional wines.

### 2.3. Wines Analyses

Wine bottles were stored in the dark and analyzed immediately after opening, without dilution. 

Total polyphenols were evaluated by the Folin–Ciocalteu’s (FC) method with gallic acid as the reference compound [14,15]. Briefly, wine samples (20 μL) were diluted with distilled water to give a final volume of 1 mL, then 0.1 mL of Folin–Ciocalteu’s reagent was added. After 5 min, 0.2 mL of sodium carbonate (35% *w*/*v*) was added. The final volume was adjusted to 2 mL with distilled water. After 1 h in the dark, absorbance at 765 nm was measured against an appropriate blank reagent. Results are expressed by reference to the calibration curve obtained with gallic acid, i.e., mg/L of gallic acid in wine.

The total flavonoids content was measured by a colorimetric method previously described, using catechin as reference compound [17,18,28]. Briefly, wine samples (0.25 mL) were diluted with distilled water to give a final volume of 1.5 mL followed by the addition of 75 μL of 5% NaNO_2_ solution. After 6 min, 150 μL of a 10% AlCl_3_ hexahydrate was added and allowed to stand for another 5 min, before 0.5 mL of 1 M NaOH was added. The mixture was adjusted to 2.5 mL with distilled water, mixed, and the absorbance measured at 510 nm. Results are expressed by reference to the calibration curve, mg/L of catechin in wine.

The total antioxidant activity of wines was measured by the Ferric Reducing Antioxidant Power (FRAP) assay [29], a colorimetric method that measures the reduction of a ferric-tripyridyltriazine complex to its ferrous colored form, in the presence of antioxidants. The reaction was monitored for 6 min after the addition of wine (10 μL) to the FRAP reagent, and the 6-min absorbance readings used for calculation. The reducing capacity of wine was calculated with reference to the ferrous sulfate using the calibration curve (range 10–100 μM). The antioxidant activity of wine is expressed as mM Fe^2+^ equivalents/L of wine.

Further, the total antioxidant activity of wines was also evaluated via the 2,2′-azino-bis (3-ethylbenzothiazoline-6-sulfonic acid) diammonium salt radical cation decolorization (ABTS) assay, which is based on free radical scavenging capacity [30]. The preformed radical monocation of 2,2′-azinobis-(3-ethylbenzothiazoline-6-sulfonic acid) (ABTS^•+^) absorbing at 734 nm is generated by the oxidation of ABTS with potassium persulfate and is reduced in the presence of hydrogen-donating antioxidants, resulting in a decolorization reaction measured as the percentage of inhibition. The ABTS radical cation was produced by reacting ABTS solution (7 mM) with potassium persulfate (2.45 mM final concentration) in distilled water at room temperature in the dark for 16 h before use. A working solution (ABTS reagent) was diluted to obtain absorbance values between 1.4 and 1.5 AU at 734 nm and prewarmed at 30 °C. After, for up to 12 min. The percentage inhibition of absorbance was calculated and the reducing capacity of wine expressed with reference to a trolox calibration curve (spanning 1–10 μM) as mM trolox equivalents/L in wine. All solutions were prepared daily. 

Total sulfur dioxide and total acidity were measured according to European official methods [31].

### 2.4. Sulfite Addition on Standard Polyphenol, Flavonoid, and Antioxidant Activity Assays

To study the effect of sulfites addition on the standard FC, flavonoids, FRAP, and ABTS methods, assays were performed (as described above), except wine was omitted and sulfites (in the range 1–20 μg) were added before reactions with each of the respective standard reference compounds were started. The standard reference compounds were gallic acid (0.5–10 μg/mL) for the FC assay, catechin (2–20 μg/mL) for the flavonoids assay, ferrous sulfate (10–100 μM) for the FRAP assay, and trolox (2–10 μM) for the ABTS assay.

### 2.5. PVPP Treatment

To separate the phenolics and non-phenolic reducing compounds (such as sulfites, sugars, ascorbic acid, and glutathione) present in the conventional wine, the water-insoluble synthetic polymer PVPP, which specifically adsorbs phenolic compounds, was used [32,33,34,35] as briefly described below. The three cycles of extraction with PVPP described by Bridi et al. allow for the almost complete adsorption of polyphenols [32].

Dry PVPP (100 mg) was added to 1 mL conventional wine. After 30 s vortexing, samples were centrifuged at 13,000× *g* for 5 min at room temperature. The supernatant (0.7 mL) was transferred and 70 mg of dry PVPP added. Samples were vortexed and centrifuged as above. The supernatant (0.5 mL) was transferred and dry PVPP (50 mg) added. Samples were vortexed and centrifuged as above. The final supernatant was analyzed for total polyphenol and flavonoid content, and for antioxidant activity, as above. The values obtained, which represent the contribution of sulfites and other non-phenolic reducing compounds to the different assays, were then subtracted from the values measured in the corresponding untreated wines.

To evaluate the efficiency of PVPP adsorption polyphenols from wine, a separate set of experiments involving four cycles of extraction of white wines with PVPP was performed. No further adsorption of polyphenols was observed compared with the three cycles of extraction procedure; therefore, the three-cycles extraction was used in this study.

### 2.6. Statistical Analysis

Data presented are means ± standard error. All experiments were performed at least in triplicate. Statistical analysis was performed by Student *t*-test, using KaleidaGraph 4.0 (Synergy Software, Reading, PA, USA). Student’s *t*-test was also used for regression analyses. The probability of *p* < 0.05 was considered statistically significant.

## 3. Results

### 3.1. Wines Characterization

The characteristics of the organic and conventional white wines are reported in Table 1.

The white wines, produced in Italy, were from 2014 or 2015 vintages. Alcoholic strength ranged from 12.5% to 13.0% for organic wines and 11.0–12.5% for conventional wines. Total acidity was 5.1–6.5 g/L for organic wines and 4.9–5.0 g/L for conventional wines. Total sulfur dioxide content was ≤3.5 mg/L for organic wines and 49.9 ± 80.2 mg/L for conventional wines.

### 3.2. Impact of Sulfite Addition on the Standard Assays Used for Measurement of Total Polyphenols, Total Flavonoids, and Antioxidant Activity

**Figure 1:**
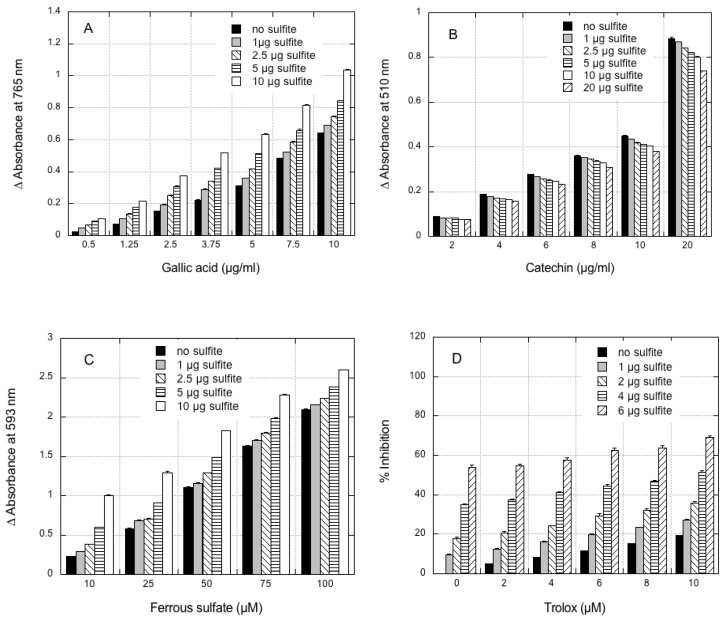
Effect of sulfite addition on the standard assays used for measuring polyphenols, flavonoids, and antioxidant activity. (**A**) Folin–Ciocalteu (FC) assay carried out with gallic acid as the reference compound; (**B**) flavonoids assay carried out with catechin as the reference compound; (**C**) ferric reducing antioxidant power (FRAP) assay carried out with ferrous sulfate as the reference compound. The reaction was followed at 593 nm; (**D**) 2,2′-azino-bis (3-ethylbenzothiazoline-6-sulphonic acid (ABTS) assay carried out with trolox as the reference compound; the percentage inhibition was determined relative to the control without trolox. Data are means of three replicates ± SE.

The effect of the addition of increasing amounts of sodium sulfite (i.e., 1–10 μg) on the reaction of gallic acid as a standard in the FC assay is shown in Figure 1A.

For each of the sulfite concentrations tested, the absorbance at 765 nm increased linearly with increased gallic acid concentrations. The extent of interference was higher at low gallic acid concentrations and decreased with increasing gallic acid levels: for the maximum sulfite level tested (i.e., 10 μg), a 4.0-fold increase in absorbance was measured at 0.5 μg of gallic acid, relative to the corresponding control, without sulfite addition, while a 1.6-fold increase in absorbance was measured at 10 μg of gallic acid. At constant gallic acid concentrations, the absorbance at 765 nm increased with increased sulfite additions (*p* < 0.01 for all sulfite levels tested relative to the control, without sulfite). At the highest gallic acid concentration, an increase of 61% was observed for the highest sulfite level tested (i.e., 10 μg), while a 7% increase was measured at the lowest sulfite concentration tested (i.e., 1 μg). No significant reaction of sodium sulfite alone was observed during the FC assay.

The influence of sulfite addition (at 1–20 μg) on the reaction of catechin during the flavonoid assay is shown in Figure 1B. A significant negative interference was observed and absorbance at 510 nm decreased with increasing additions of sodium sulfite for all catechin concentrations tested relative to the control without sulfite addition (*p* < 0.05 for all sulfite concentrations tested, except for 1 μg sulfite at the highest catechin level). For the maximum sulfite level (20 μg), the absorbance at 510 nm significantly decreased to about 84% of the control for the highest catechin concentration tested, and to about 90% of the control for the lowest catechin level tested. No significant reaction of sodium sulfite was observed during the flavonoid assay.

The effect of sulfite addition (<8 at 1–100 μM) on the reaction of standard ferrous sulfate (Fe^2+^) in the FRAP assay is shown in Figure 1C. A strong dose-dependent, positive interference of sulfite was observed (*p* < 0.02 for all sulfite concentrations tested). For each sulfite concentration, the absorbance at 593 nm showed a linear increase with increased ferrous sulfate concentrations (*p* < 0.02 for all the sodium sulfite levels tested). The extent of the interference was higher at low ferrous sulfate concentrations, and decreased with increasing ferrous sulfate levels: for the maximum sulfite level tested (10 μg), a 4.5-fold increase in absorbance was measured at 10 μM ferrous sulfate, relative to the control without sulfite addition, while a 1.2-fold increase in absorbance was measured at 100 μM ferrous sulfate.

Figure 1D shows the effect of sulfite addition on the ABTS radical cation decolorization assay using the antioxidant trolox as a reference compound. For each sulfite concentration tested, the percentage inhibition, which reflects the scavenging ability, showed a linear increase with increased trolox concentrations (*p* < 0.0001 for all sulfite concentrations tested). The extent of the interference decreased when trolox concentrations were increased: for the maximum sulfite level tested (6 μg), an 11.5-fold increase in percentage inhibition was measured at 2 μM trolox compared with the corresponding control without sulfite addition, while a 3.5-fold increase in percent inhibition was measured at 10 μM of trolox.

**Figure 2:**
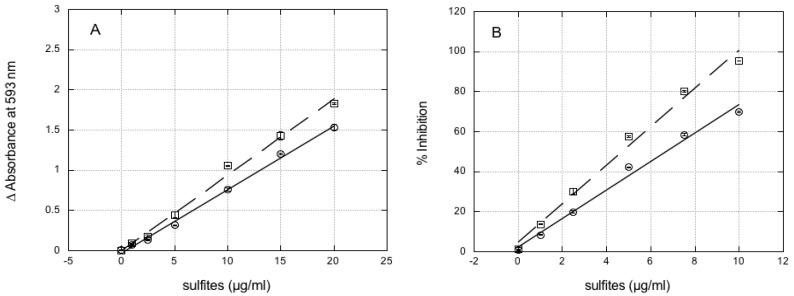
Reaction of sodium sulfite and sodium metabisulfite in the FRAP and ABTS assays. FRAP (**A**) and ABTS (**B**) assays were carried out with increasing amounts of either sodium sulfite (open circles) or sodium metabisulfite (open squares). Data are means of three replicates ± SE.

Very similar results were obtained when sodium sulfite was replaced with sodium metabisulfite for all of the assays (i.e., FC, flavonoids, and antioxidant activity assays). Neither sodium sulfite or sodium metabisulfite significantly reacted during the FRAP assay (Figure 2A) and in the ABTS assay (Figure 2B).

A linear relationship was observed between sulfite/metabisulfite amounts and either absorbance at 593 nm in the FRAP assay (*r* = 0.998, *p* < 0.0001 for sulfite, *r* = 0.997, *p* < 0.0001 for metabisulfite) or percentage inhibition in ABTS assay (*r* = 0.994, *p* < 0.0001 for both sulfite and metabisulfite), in the concentration range tested.

### 3.3. Total Polyphenols, Flavonoids, and Antioxidant Activity in White Wines

To study the effect of sulfite additions on white wine analyses, three organic white wines, made without any sulfite addition during winemaking, were sourced. Increasing amounts of sulfites (either sodium sulfite or sodium metabisulfite) were added to the organic wines, which were then analyzed for polyphenols and flavonoids content and antioxidant activity.

**Figure 3:**
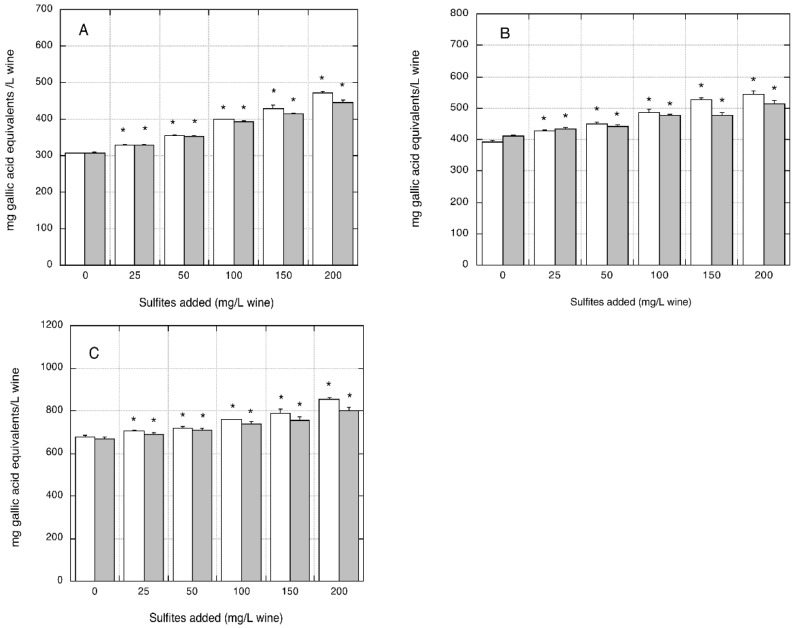
Effect of sulfite/metabisulfite addition to organic white wines on polyphenols measurements. FC assay was performed using gallic acid for the calibration curve. (**A**) AO organic wine; (**B**) BO organic wine. (**C**) CO organic wine. Closed grey bars: sodium sulfite addition. Open white bars: sodium metabisulfite addition. Data are means of four replicates ± SE. * *p* < 0.05 relative to control wine without sulfite/metabisulfite addition by Student *t*-test.

As shown in Figure 3A–C, the addition of increasing amounts of sodium sulfite or sodium metabisulfite to three different organic white wines resulted in a significant and dose-dependent interference in the FC assay.

The content of gallic acid measured increased linearly with increasing sulfite additions to the wine (*p* < 0.005 for all sulfite concentrations tested). At the maximum sulfites level (i.e., 200 mg/L), the increases in the polyphenols content of the three wines were +44.4%, +25.2%, and +29.1% for sodium sulfite and +53.9%, +39.1%, and +32.6% for sodium metabisulfite, respectively, compared with the corresponding control wines (*p* < 0.05).

**Figure 4:**
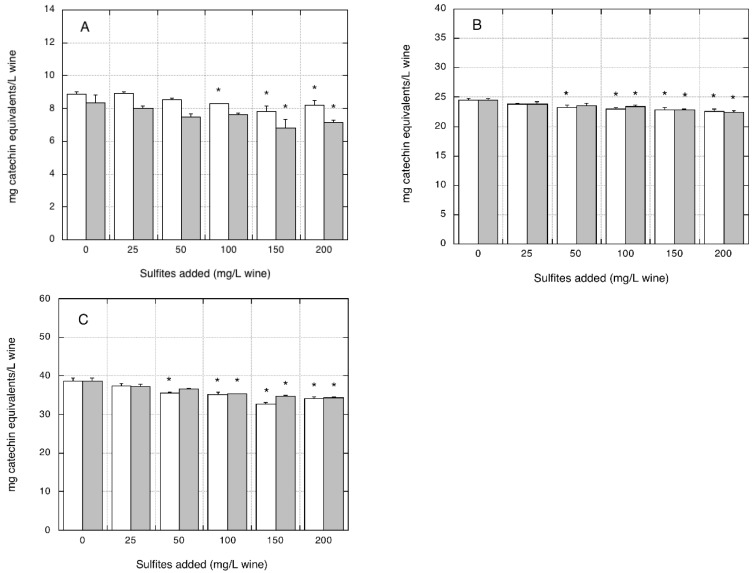
Effect of sulfite/metabisulfite addition to organic white wines on flavonoids measurements. Flavonoids assay was performed using catechin for the calibration curve. (**A**) AO organic wine. (**B**) BO organic wine. (**C**) CO organic wine. Closed grey bars: sodium sulfite addition. Open white bars: sodium metabisulfite addition. Data are means of four replicates ± SE. * *p* < 0.05 relative to control without sulfite/metabisulfite addition by Student *t*-test.

With regard to flavonoids measurements, the addition of increasing amounts of sodium sulfite or sodium metabisulfite to the three organic wines resulted in a negative interference. The flavonoids content of wines decreased in a linear fashion with increasing additions of either sodium sulfite or sodium metabisulfite (Figure 4A–C).

At the lowest sulfites concentration (i.e., 25 mg/L), the observed decreases were not statistically significant. For the highest sulfite levels added (i.e., 200 mg/L), significant decreases in flavonoid content were measured in all of the three wines: −14.2%, −8.4%, and −11.1% for sodium sulfite, and −7.7%, −7.8%, and −11.8% for sodium metabisulfite, respectively, compared with the corresponding control wine without sulfite/metabisulfite addition (*p* < 0.05).

**Figure 5:**
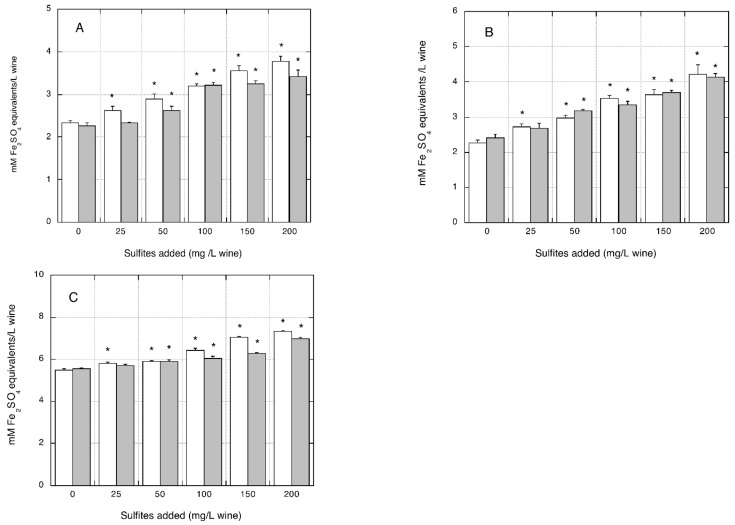
Effect of sulfite/metabisulfite addition to organic white wines on antioxidant activity measured with the FRAP assay. The FRAP assay was performed using ferrous sulfate for the calibration curve. (**A**) AO organic wine; (**B**) BO organic wine; (**C**) CO organic wine. Closed grey bars: sodium sulfite addition. Open white bars: sodium metabisulfite addition. Data are means of four replicates ± SE. * *p* < 0.05 relative to control, without sulfite/metabisulfite addition, by Student *t*-test.

Figure 5A–C shows the effects of sulfite/metabisulfite addition on antioxidant activity measurements obtained with the FRAP assay in the three organic white wines tested.

**Figure 6:**
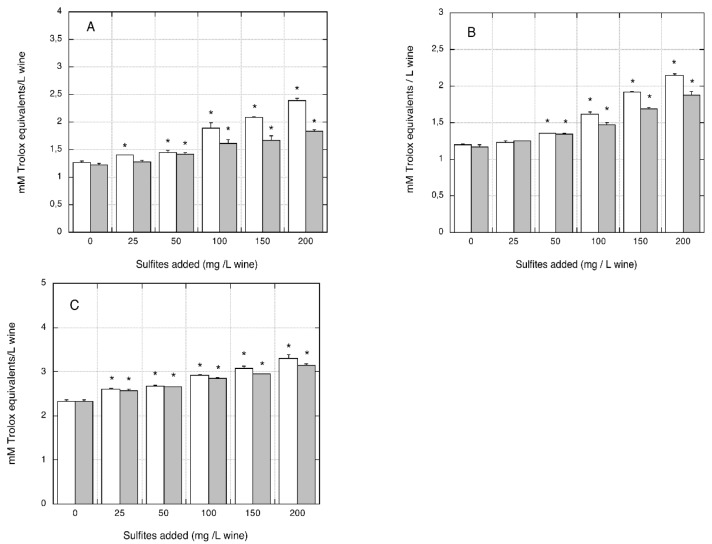
Effect of sulfite/metabisulfite addition to organic white wines on the antioxidant activity measured with the ABTS assay. The ABTS assay was performed using trolox for the calibration curve. (**A**) AO organic wine; (**B**) BO organic wine; (**C**) CO organic wine. Closed grey bars: sodium sulfite addition. Open white bars: sodium metabisulfite addition. Data are means of four replicates ± SE. * *p* < 0.05 relative to control, without sulfite/metabisulfite addition, by Student *t*-test.

The antioxidant activity increased linearly with increased sulfite/metabisulfite additions. A significant overestimation of antioxidant activity was observed for sulfite additions in the range of 50–200 mg/L (being +50.6%, +70.9%, and +25.0% for the highest sulfite level tested) and for metabisulfite additions in the range of 25–200 mg/L wine (+62.5%, 87.2%, and 33.5% for the highest metabisulfite level tested) in the three organic wines (*p* < 0.05 compared with the corresponding control wine without sulfite/metabisulfite addition). When antioxidant activity was measured with the ABTS assay, a similar trend was observed in all three organic wines tested. As shown in Figure 6A–C, a significant overestimation of antioxidant activity was measured by the ABTS assay following sulfite/metabisulfite addition in the range of 50–200 mg/L. At the highest additive rate (i.e., 200 mg/L), the increased antioxidant activity measured in the organic white wines was +49.5%, +60.4%, and +35.4% with sulfite addition and +89.3%, +78.8%, and +41.6% with metabisulfite addition, (*p* < 0.05 relative to the corresponding control wines without sulfite/metabisulfite addition).

### 3.4. Effect of PVPP Treatment of Conventional Wines on Measurement of Total Polyphenols, Flavonoids, and Antioxidant Activity

To account for the interference of sulfites (and other possible non-phenolic reducing compounds) on polyphenol, flavonoid, and antioxidant activity measurements, three conventional white wines (containing 60.5, 49.9, and 80.2 mg/L of total sulfur dioxide, Table 1) were pretreated with PVPP, which specifically absorbs polyphenols [32,33,34,35]. For each wine, the assays were performed on both the untreated wine and on the final supernatant obtained after PVPP pretreatment of wine, that is, wine deprived of polyphenols, representing the contribution of sulfites and other non-phenolic reducing compounds to the various assays. The values obtained with the untreated wine were subtracted from the values measured in the corresponding supernatant obtained after PVPP treatment.

**Figure 7:**
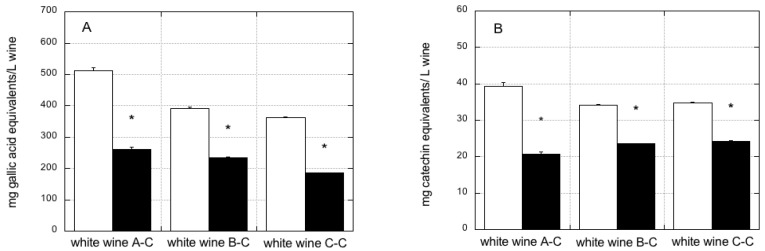

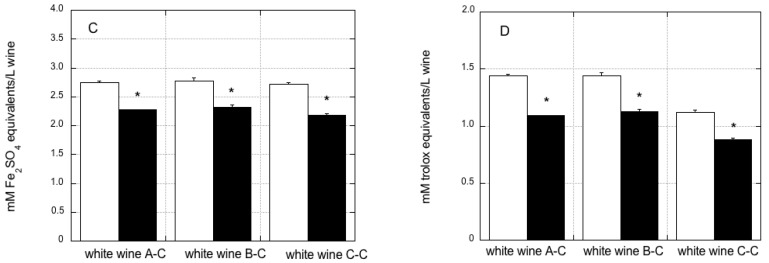
Effect of PVPP treatment on polyphenol, flavonoid, and antioxidant activity measurements of three conventional white wines. Polyphenols content (**A**), flavonoids content (**B**), and antioxidant activity were measured with the FRAP assay (**C**) and the ABTS assay (**D**). Open white bars: measurements were performed on untreated wines. Closed grey bars: values measured for untreated wine were subtracted from the values measured in the corresponding supernatant obtained after PVPP treatment. This represents the contribution of sulfites and of other non-phenolic reducing compounds. Data are means ± SE (*n* = 6). * *p* < 0.0001, relative to the untreated wines, by Student *t*-test.

Figure 7 shows the effect of PVPP treatment on total polyphenols, total flavonoids, and antioxidant activity measurements (FRAP and ABTS assays) in the three conventional white wines.

The total polyphenol content of the three conventional white wines was obtained by subtracting the contribution of sulfites and other non-phenolic reducing compounds (from the final supernatant obtained after PVPP treatment of wine). This was significantly lower (51.1%, 60.0%, and 51.0%, *p* < 0.0001) in comparison to the levels measured in the respective untreated wine (Figure 7A). Similar results were observed for total flavonoids and antioxidant activity measurements. By subtracting the contribution of sulfites and other non-phenolic reducing compounds, the total flavonoids content of the three conventional white wines was 52.5%, 69.0%, and 69.9% of that measured in the respective untreated wines (*p* < 0.0001) (Figure 7B), suggesting that, in addition to sulfites, other interfering non-phenolic reducing compounds are present in conventional white wines. As with regard to the antioxidant activity measurements, by subtracting the contribution of sulfites and other non-phenolic reducing compounds, the FRAP values of the three conventional white wines were 82.6%, 83.7%, and 80.4% of those measured in the respective untreated wines (*p* < 0.0001) (Figure 7C), while the ABTS values were 75.9%, 78.3%, and 78.6% of those measured in the untreated wines (*p* < 0.0001) (Figure 7D). Therefore, for all three conventional white wines, the measurements obtained after PVPP treatment for total polyphenols, flavonoid content, and antioxidant activity were significantly lower compared with untreated wines.

## 4. Discussion

In this study, we used both sodium sulfite and sodium metabisulfite to test the effects of sulfites on antioxidant activity, and total polyphenol and flavonoid measurements, both in standard assays and in white wine analyses.

Firstly, our data clearly show a significant interference of sulfites, when added to the standard assays either as sodium sulfite or sodium metabisulfite, during the use of analytical methods for determination of antioxidant activity (FRAP and ABTS assays), polyphenols (FC assay), and flavonoids (aluminium-chloride assay). In particular, the positive interference observed for the FC, FRAP, and ABTS assays will lead to remarkable overestimation of polyphenols content and antioxidant activity measurements, while negative interference was observed for the flavonoids assay, which will result in the underestimation of flavonoids. These interfering effects occur at sulfites levels close to those present in wines. Neither sulfite nor metabisulfate alone reacted in the FC and flavonoids assays in the concentrations range tested, while both of them reacted with FRAP and ABTS reagents in the respective assays.

With regard to white wine analyses, data presented here demonstrate that the addition of sulfites, either as sodium sulfite or sodium metabisulfite, to organic white wines actually results in a significant overestimation of the total polyphenols content and antioxidant activity of wine, while in the presence of sulfites, the flavonoids content may be significantly underestimated, as predicted by the results obtained in the standard assays. Our results are in agreement with some previous observations on polyphenols measurements with the FC assay in white wine [23,25]. An overestimation of the antioxidant potential measured with the DPPH assay was also observed in white wine [25].

Somers and Ziemelis reported that total polyphenols measurements by FC assay may be magnified several-fold by sulphur dioxide and suggested that the interference is due principally to a synergistic reaction between sulphur dioxide and *o*-dihydroxy phenols [23]. A similar synergistic mechanism has been proposed by Saucier and Waterhouse [36] between catechin and sulphur dioxide in FC assay and metmyoglobin assay. Although the mechanism of the synergy observed with sulphur dioxide remains unknown, the authors hypothesized that the phenolic quinone (oxidation product) may be recycled back to the phenol by the oxidation of sulfite to sulfate. Such a mechanism could explain the magnification effect of sulfites, not only in the FC assay, but also in the FRAP and ABTS assays, observed in this study. The mechanism of the detrimental effect of sulfites on total flavonoids measurements is unknown. It may be hypothesized that sulfites, when present at high concentrations, may interfere with aluminium complexation by flavonoids.

With regard to the total polyphenols measurements, some solutions have been proposed to overcome sulfites interference. To measure the total polyphenols content of white wine, it has been proposed that the total sulfites should be determined and the contributions of the polyphenols calculated by subtraction [25]. Alternatively, the polyphenols content of wine could be determined in an enzymatic, peroxidase-catalyzed assay, where the contribution of the interfering substances, such as sulfites, is less pronounced [24]. Somers and Ziemelis suggested a direct spectrophotometric procedure using absorbance at 280 nm or qualitative and quantitative HPLC analyses to measure wine polyphenols [23].

Recently, a PVPP-based procedure has been applied to evaluate total phenolics with the FC assay in beverages (fruit juices, tea, and wine) by Bridi et al. [32]. PVPP is an water-insoluble, synthetic polymer often used by industry to remove haze-active polyphenols from beer, apple juice, and wine [33,34,35]. In their study, Bridi et al. used a three-cycles extraction process with PVPP to separate phenolics from non-phenolic reducing compounds (e.g., sulfites, sugars, ascorbic acid, glutathione) in beverages [32]. This procedure allows for the almost complete adsorption of polyphenols [32]. In our study, we took advantage of this PVPP-based procedure to assess antioxidant activity and total polyphenols and total flavonoids content in conventional white wines (i.e., wines containing added sulfites). The adsorption and removal of polyphenols from wine by the PVPP procedure allowed for the quantification of the contribution of sulfites and other non-phenolic reducing compounds to each assay. Our data show that the total polyphenols and flavonoids content and antioxidant activity measurements obtained after applying the PVPP-based procedure to conventional wines were all significantly lower compared to those measured in the corresponding untreated wines. Sulfites addition to the standard flavonoids assay results in a negative interference (Figure 1B). Accordingly, sulfites addition to organic white wine (wine produced without sulfites addition during the winemaking procedure) results in a negative interference on the flavonoids assay (Figure 3B). Therefore, the removal of sulfites from conventional white wine (wine produced with the addition of sulfites during winemaking) would be expected to give higher values for flavonoid content. However, our results show that, after the removal of sulfites and other non-phenolic reducing compounds by PVPP treatment, the flavonoids content of conventional wine is lower than that measured in untreated wine, suggesting that, in addition to sulfites, other non-phenolic reducing compounds are present in wine and also interfere with the flavonoids assay.

## 5. Conclusions

From our data, it can be concluded that the direct analysis of polyphenols, flavonoids, and antioxidant activity of conventional white wines may result in a lack of accuracy of the parameters measured. Therefore, a purification procedure for white wine is needed. The use of a PVPP-based procedure, which almost completely adsorbs polyphenols, might offer a useful tool to quantify the contribution of sulfites and other non-phenolic compounds to each assay and to obtain a more reliable estimation of the total polyphenols and flavonoids content and antioxidant activity of white wine. Further, accurate studies involving the addition of sulfate to other beverages are needed to verify the possible interferences from sulfites on their nutritional quality. Finally, the feasibility of alternative purification procedures should be also explored.

## Figures and Tables

**Table 1:** Characteristics of the organic and conventional white wines.

Wine Code	Grape Variety	Vintage	Alcohol (%)	Total Acidity ^1^ (g/L)	Total Sulfur Dioxide ^2^ (mg/L)
Organic wines ^3^:					
A-O	Cortese di Gavi	2014	12.5	5.1	≤3.5
B-O	Chardonnay, Lison	2014	12.5	5.3	≤3.5
C-O	Friulano (Tocai)	2014	13.0	6.5	≤3.5
Conventional wines:					
A-C	Trebbiano	2014	11.5	4.9	60.5 ± 3.6
B-C	Trebbiano	2015	12.5	5.0	49.9 ± 1.8
C-C	Trebbiano Rubicone	2014	11.0	5.0	80.2 ± 3.6

^1^ Total acidity values are expressed as tartaric acid equivalents. Values are means of three determinations (*n* = 3), (standard error (SE) ≤0.15); ^2^ Values are mean of three determinations ± SE (*n* = 3); ^3^ Organic wines were produced without sulfur dioxide/sulfites addition.
